# *Withania somnifera* L.: Insights into the phytochemical profile, therapeutic potential, clinical trials, and future prospective

**DOI:** 10.22038/IJBMS.2020.44254.10378

**Published:** 2020-12

**Authors:** Sumaira Saleem, Gulzar Muhammad, Muhammad Ajaz Hussain, Muhammad Altaf, Syed Nasir Abbas Bukhari

**Affiliations:** 1 Department of Chemistry, GC University Lahore, Lahore 54000 Pakistan; 2 Department of Chemistry, University of Sargodha, Sargodha 40100, Pakistan; 3 Department of Pharmaceutical Chemistry, College of Pharmacy, Jouf University, Aljouf, Sakaka2014, Saudi Arabia

**Keywords:** Clinical trials, Folk medicinal uses, Nutraceuticals, Pharmacological attributes, Phytonutrients, Withanolides

## Abstract

*Withania somnifera* L. is a multipurpose medicinal plant of family *Solanaceae *occurring abundantly in sub-tropical regions of the world. The folk healers used the plant to treat several diseases such as fever, cancer, asthma, diabetes, ulcer, hepatitis, eyesores, arthritis, heart problems, and hemorrhoids. The plant is famous for the anti-cancerous activity, low back pain treatment, and muscle strengthening, which may be attributed to the withanolide alkaloids. *W. somnifera* is also rich in numerous valued secondary metabolites such as steroids, alkaloids, flavonoids, phenolics, saponins, and glycosides. A wide range of preclinical trials such as cardioprotective, anticancer, antioxidant, antibacterial, antifungal, anti-inflammatory, hepatoprotective, anti-depressant, and hypoglycemic have been attributed to various parts of the plant. Different parts of the plant have also been evaluated for the clinical trials such as male infertility, obsessive-compulsive disorder, antianxiety, bone and muscle strengthening potential, hypolipidemic, and antidiabetic. This review focuses on folk medicinal uses, phytochemistry, pharmacological, and nutrapharmaceutical potential of the versatile plant.

## Introduction

Currently, researchers are more captivated by plant-based bioactives, which provide health benefits owing to the presence of high-value secondary metabolites. In the current world healthcare scenario, more than 80% of the world population, particularly in developing countries, trusts phytomedicines for the therapeutic needs (1). Still prevailing success of traditional systems of therapies such as Ayurveda, Greco-Arab (Unani-Tibb), and Chinese medicine system is due to the fact that phytomedicines have fewer side effects and are economical (2-4).

**Figure 1:**
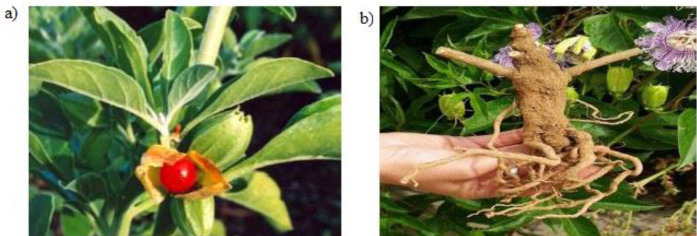
a) The plant with cherry and leaves b) The roots of W. somnifera (reprinted from reference (3) with permission of publisher Global Research Online, Bangalore, India)

The plants belonging to the genus* Withania *of *Solanaceae* family are generally acclaimed as medicinally essential due to the high therapeutic and nutraceutical potential. One of the plants of the genus, *W. somnifera *(Figure 1)*,* possessed a plethora of medicinal uses and pharmacological applications. *W.* *somnifera* (Syn: ashwagandha, suranjan, winter cherry, Indian ginseng) is a xerophytic plant that nurtures abundantly in Africa, the Mediterranean, Sri Lanka, Pakistan, and India (5-9).

In the Ayurvedic system of medicines, roots and leaves of the plant were considered phytotherapeutic agents to cure various ailments. Various clinical and preclinical trials exhibited the plant’s potential in curing hepatotoxicity (10), neurological disorders (11), anxiety (12), Parkinson’s disease (13), and hyperlipidemia (4, 14). The fruits contained considerable amounts of saponins and leaves possessed insect repellent properties (15).

Phytochemical analysis of *W. somnifera* revealed the presence of pharmacologically active steroidal lactones named withanolides (16, 17). Withanine, a group of alkaloids isolated from the roots of the plant, forms 38% of the total weight of alkaloids (18). The principal withanolides extracted from *W. somnifera* in India were withanolide D and withaferin A which exhibited antitumor and cytotoxic properties (19). In addition to alkaloids, the plant also consisted of steroids, saponins, phenolics, flavonoids, phytophenols, and glycosides (20-23). Also, it is widely used in traditional medicine formulations as an antipyretic, analgesic, adaptogenic, and anti-inflammatory agent (5, 24).

Literature has witnessed a few review articles discussing some other aspects of *W. somnifera *(25, 26). However, a comprehensive review is still in demand to appraise state of the art concerning phytochemical composition, medicinal applications, and nutrapharmaceutical potentials of the miraculous plant *W. somnifera*. We emphasized mainly the clinical trials, toxicity, and pre-clinical trials of the multiuse plant. This review will help to bridge the knowledge gaps among pharmacists, medicinal chemists, and pharmacologists about phytochemicals, the therapeutic uses, and pharmaceutical applications of the beneficial plant. The review appraised recent papers on the high value-added applications of the plant material in medicine and pharmacy.

**Phytochemistry**

The plants are rich in phytochemicals such as alkaloids, steroids, terpenoids, etc., which are important parts of food and folk medicine in the history of mankind (27-30). Power and Salway initiated phytochemical studies of *W. somnifera* in 1911 with the isolation of withaniol, somnirol, somnitol, withanic acid, phytosterol, ipuranol, and alkaloids from alcoholic extracts of leaves and roots (31). Alkaloids isolated in the study mentioned above were named as somniferine, somnine, somniferinine, withamine, withanmine, pseudowithamine, and withanaminine (32). It was investigated that alcoholic extract of the plant contains various phytochemicals such as tropine, choline, pseudotropine, *dl*-isopelletierine, cuscohygrine, anahygrine, and anaferine (33). Furthermore, a pyrazole alkaloid, withasomnine, was separated from the alcoholic root extract of *W. somnifera* (34). In 1980 the presence of tisopelletierine, 3α-tigloyloxtropine, cuscohygrine, 3-tropyltigloate, hygrine, *dl*-isopelletierine, withasomnine, mesoanaferine, withanine, somniferine, hentriacontane, withananine, visamine, ashwagandhine, and pseudowithanine in methanolic extract of the plant leaves was reported (35). Besides, the methanolic extract of the plant was shown to have withaniol, reducing sugars, acylsteryl glucosides, ducitol, starch, hantreacotane, iron, and amino acids such as aspartic acid, proline, tyrosine, alanine, glycine, glutamic acid, cysteine, and tryptophan (20, 21, 36). Seven new withanosides I-VII were obtained from methanolic extract of the plant root, and structures were confirmed using Fast Atom Bombardment-Mass Spectrometry, ^13^C and ^1^H nuclear magnetic resonance (NMR), and UV-Visible spectroscopic techniques (21).

Methanolic extract consisted of steroidal lactones named as withanolides (37-39). Withanolide D isolated from the alcoholic extract of leaves exhibited structural similarity to withaferin A except for the hydroxyl group at C-20 instead of C-27 (39). Withanolides forming 0.001-0.5% of the total dry weight of leaves and roots (40) are actually 22-hydroxyergostane-26-oic-26,22-olide with novel structural variants at a carboxylic skeleton or side chains (41). Distinct chemotypes of *W. somnifera* consisted of different quantities of substituted steroidal lactones depending upon geographical distribution (15, 16, 32, 39, 42). Presence of 4β,27-dihydroxy- 5β,6β-epoxy-1-oxowitha-2,24-dienolide skeleton in chemotype I was the result of chemo-genetic variations in *W. somnifera* while chemotype II contained excessive concentrations of withanolide D (35,43). Withanolide D was mainly observed in chemotype-II, which was featured by structural modification at C-20 containing a hydroxyl group at 4β and epoxy system at 5 and 6β positions. Structural characterization revealed that withanolides E and J contained an OH group at C-17, not at C-20, unlike other withanolides. Withanolides E and F showed α-orientation while withanolides G and J possessed normal β-orientation of side chains (44). The substituted steroidal lactones were characteristics of each chemotype evolved from genetic variations (36) and extensive study of the chemotypes showed variable steroidal contents (45-50).

Withanolide A isolated from the alcoholic extract of roots (51) was characterized as 4β,27-dihydroxy-1-oxo-5β,6β-epoxy with a-2,24-dienolide (52). Similarly, withanolide C, isolated from the plant, *possessed *5 and 6β epoxide rings with chloro-group at C-5 (53). Various sitoindosides were produced by substitution of the acyl group (sitoindoside VII and VIII) and glucose units (sitoindoside IX and X) at C-27 of saponins. Withanone and tubacapsenolide F, with six derivatives, were isolated from aqueous extract of the whole plant (54). Spectroscopic studies revealed the presence of 2-en-1-one with a steroid having an epoxy group at 6 and 7 positions, which was verified by 2-mercaptoethanol with 5 and 6β epoxy steroids (55). Withanolide Q consisted of a hydroxyl group at C-23 while OH group was absent at C-27 in withanolide R (56). Withanolide S isolated from the alcoholic extract of leaves during biogenetic study possessed close structural similarity to withanolide E, except the presence of secondary axial hydroxyl moiety with lack of epoxide ring. Two novel withanolides N and O (45) along with known withanolide D, E (16, 39), and P (57) were discovered from the alcoholic extract of plant leaves. Another study displayed the presence of eight novel withanolides F–M from the alcoholic extract of air-dried crushed leaves (17, 57).

Quantitative analysis of leaves revealed that withaferin A is 1.6% of total dry weight (58). Leaves of the plant were extracted with ethanol and investigated to contain (5R,6S,7S,8S,9S,10R,13S,14S,17S,20R,22R)-6,7α-epoxy-5,17-α,27-trihydroxy-1-oxo-22R-witha-2,24-dienolide (59). Several dragendorff positive alkaloids isolated from crude methanolic extract of roots were recognized as pseudotropine, cuscohygrine, isopelletierine, *dl*-isopelletierine-3-tropyltigloate, anaferine, hygrine, anahygrine, somniferine, meso-anaferine, 3α-tigloyloxtropine, choline, withanine, visamine, withananine, hentriacontane, and withasomnine along with pyrazole derivatives, pseudowithanine, and ashwagandhine. It was concluded, using spectral studies, that methanolic extract of shade-dried aerial parts possessed chlorinated withanolide (27-acetoxy-4*β*,6*α*-dihydroxy-5*β*-chloro-1-oxowitha-2,24-dienolide) along with diepoxy withanolide, and withaferin A, which displayed anti-cancerous potential against human lung cancer (NCI-H460 cell line) (60). Steroidal lactones, withanolide G and Δ16-withanolide (61-63) were isolated from chemotype III. Whole plant extracted with equimolar ratios of water and methanol was characterized using spectroscopic techniques and X-ray crystallographic analysis and explored and found to contain new chlorinated withanolide, 6α-chloro-5β,17α-dihydroxywithaferin A along with nine known withanolides such as (22R)-5β-formyl-6β,27-dihydroxy-1-oxo-4-norwith-24-enolide, 6α-chloro-5β-hydroxywithaferin A, 2,3-dihydrowithaferin A, withaferin A, withanone, 3-methoxy-2,3-dihydrowithaferin A, withanoside IV, 2,3-didehydrosomnifericin, and withanoside X. Ethanol extract of the plant was examined and shown to have withasomniferin A, iso-sominolide and sominone (64, 65)*.*  Leaves of crossbreed of *W. somnifera* from Israel and new Dehli (India) regions were extracted with ethanol and showed the presence of withanolide T and U (46, 57, 66, 67).

Withasomidienone, isolated from the methanolic root extract of the plant, showed three double bonds at 1, 4, and 24 positions, which are a characteristic feature of most of the withanolides (68). In another investigation, withaoxylactone and somnifericin were isolated from the plant and characterized using various techniques. It was disclosed that four epoxy groups are present at different positions of withaoxylactone (5, 6, 14, and 15 positions) and somnifericin (4, 5, 6, and 27 positions) (69).

Another investigation revealed the presence of five novel withanolides such as withasomnilide, somniferanolide, somniferawithanolide, withasomniferanolide, and somniwithanolide in ethanolic extract of stem bark. Further examination showed that epoxy groups are not present in withasomniferanolide, somniferawithanolide, and somniwithanolide (70). Similarly, three new withanolides such as withasomniferol A, withasomniferol B, and withasomniferol C were isolated from ethyl acetate and benzene extracts of roots (71). 4-Deoxywithaperuvin isolated from alcoholic fruit extract was characterized by spectral analysis and found to contain five hydroxyl moieties (at 5, 6, 14, 17, and 20 positions) (72). Viscosalactone B in the alcoholic extract of the whole plant was investigated as a structural analog of withaferin A having an epoxy group at 6 and 7 positions, double bond at C- 24, and three hydroxyl groups at 3, 4, and 27 positions of carbon skeleton (73). Withanolide Y was identified as 5α, 6α-epoxy-7α, 17α, 20R-trihydroxy-1-oxo-22R-witha-2, 24-dienolide using single-crystal XRD (74). Extraction of plant’s root with petroleum ether and acetone was carried out for the isolation of phytochemicals. Various chromatographic and spectroscopic techniques depicted the presence of β-sitosterol and *d*-glycoside (75). Subcritical water extraction of withanolides was conducted for 50 g powder of *W. somnifera*/500 ml of distilled water at 40 °C. The extract was lyophilized and withanolides were separated by soxhlet and high-performance liquid chromatography. Results showed that withanoside IV, 12-Deoxywithastramonolide, withaferin A, and withanolide A were the actual biological contents responsible for various biological activities (76). Phytochemical extraction of caffeic, ferulic, and benzoic acids along with withaferin A, withanone, and withanolide A was done from methanolic and chloroform extracts of root, stem, and leaves of *W. somnifera* at a concentration of 4.0 g of plant part (77). Chemical names, structures, and biological activities of essential withanolides are listed in Table 1. 

Different oil extracts from berries of *W. somnifera* were analyzed using GC-MS showing saturated and unsaturated fatty acids like linoleic acid (11.247%), palmitic acid (2.842%), linoleic acid (4.000%), tetracosanoic acid (0.880%), palmitic acid (0.42%), elaidic acid (0.01%), linoleic acid (0.23%), and oleic acid (0.14%) (95). Examination showed that extracts consisted of nine withanolides such as 27-hydroxy withanone, 17-hydroxy-27-deoxy withaferin A, 17-hydroxy withaferin A, withanolide D, withaferin A, withanolide A, 27-hydroxy withanolide B, withanone, and 27-deoxywithaferin A (77). Butanol extract of roots also displayed the existence of withanoside IV, physagulin, and withanoside VI (96, 97).

Methanolic extract of the fruits was explored and possessed withanamides A-I (98). In a similar study, methanolic extract of fresh berries showed the presence of 6,7α-epoxy-1α,3β,5α-trihydroxy-witha-24-enolide (99). Another study disclosed the roots of the plant were composed of novel dimeric thiowithanolide, ashwagandhanolide. A novel dimeric withanolide with unusual thioether linkage from methanolic extract of roots displayed activity against human gastric, colon (HCT-116), and lung (NCI H460) cancer, central nervous system (SF-268), and breast cancer cell lines (MCF-7) with IC_50_ range of 0.43-1.48 *µ*g/ml (100). Diaion HP-20 column chromatography was used to quantify different glycosidic fractions in the methanolic extract of plant roots. Results showed the presence of withanosides I (0.0020%), withanosides II (0.012%), withanosides III (0.0024%), withanosides IV (0.048%), withanosides V (0.017%), withanosides VI (0.024%), and withanosides VII (0.0011%) with withaferin A, 5,20α(R)-dihydroxy-6*,*7α -epoxy-1-oxowitha-2,24-dienolide, physagulin D, and coagulin Q (101). Other important phytochemicals such as 3α-(Uracil-1-yl)-2,3-dihydrowithaferin A, 2,3-dihydrowithaferin A-3β-O-sulfate, 3β-*O*-Butyl-2,3-dihydrowithaferin A, and 3β-(Adenin-9-yl)-2,3 dihydrowithaferin A were isolated from methanolic extracts of the aeroponically grown plant (102, 103).

Various earlier phytochemical investigations showed the presence of steroidal lactones, alkaloids, saponin, flavonoids, tannin, starch, phenolic content, carbohydrate, withanolides, sitoindosides, anaferine, anahygrine, β-sitosterol, chlorogenic acid, cysteine, cuscohygrine, pseudotropine, withanine, scopoletin, withananine, somniferinine, somniferiene, tropanol, 14-α-hydroxywithanone, and 6,7β-Epoxywithanon (33, 40, 52, 68, 84, 104-111). Table 2 shows vital phytochemicals from different parts of the plant.

**Folk medicinal uses**

Different parts of the plant, such as leaves, roots, flowers, bark, and stem, are traditionally used to cure heart problems, pain, liver disorders, fever, respiratory infections, wounds, ulcers, and sex-related diseases (112, 113). The curing potential of the plants used in ancient systems of medicines (Unani and Ayurvedic) owed to bioactives such as alkaloids, steroids, phenolics, flavonoids, etc., (2, 113). Among other plants of genus *Withania*, *W. somnifera* is rich in aforesaid bioactives making it the first choice of folk healers (114-117). The summary of folk medicinal uses of various parts of the plant is displayed in Table 3.

**Pharmacological attributes**

***Antimicrobial and antifungal activities***

The plant also exhibited bursal disease virus inhibition in cytopathic effect reduction assay (117). Monomeric glycoprotein isolated from root tuber of the plant inhibited the growth of fungi such as *Fusarium oxysporum, Aspergillus flavus,* and *Fusarium verticilloides*, and bacterium such as *Clvibacter michiganensis *subsp.* michiganensis* (123). Aqueous and alcoholic root extracts were examined for bactericidal potential using agar well diffusion assay. Butanolic sub-fraction of methanolic root/leaf extract of the plant exhibited significant antibacterial potential against *Salmonella typhimurium* while oral administration of aqueous leaf/ root extract of *W. somnifera* exhibited bacteriostatic effect against *S. typhimurium* similar to chloramphenicol, the standard drug (124). Different leaf, root, and stem extracts of the plant were investigated for bactericidal activity against six bacterial strains, e.g., *Staphylococcus aureus*, *Bacillius subtilis*, *Escherichia coli*, *Raoultella planticola*, *Pseudomonas aeruginosa*, *Enterobacter aerogens*, and fungistatic action against *Candida albicans* and *A. flavus *using serial dilution and disc diffusion methods. Among the extracts, aqueous leaf extract showed the highest activity against *R. planticola, * while all others showed moderate antibacterial and fungistatic activities (150-152). 

Various extracts and pure compounds obtained from the plant acted as potential antibacterial and antifungal agents (164). In another investigation, leaves and roots of the plant were extracted with methanol, diethyl ether, and *n*-hexane and assessed for antibacterial potential against *S. typhimurium* and *E. coli* using agar plate diffusion assay. The methanol and hexane based extract showed substantial antimicrobial effects. A considerable rise in antibacterial activity of Tibrim was observed on co-administering with methanolic and hexane extracts of the plant (165).

Methanolic extract of roots inhibited the growth of *E. coli* and *Enterococcus *species at a concentration of 10 μg/ml (97)*. *The methanolic extract of roots, leaves, and bark inhibited the growth of Gram-positive (*S. aureus*) and negative (*E. coli*) bacterial strains at the concentration of 10-100 μg/ml. Methanoic extracts of roots and leaves were assessed against various fungi such as *A. flavus, D. turcica*, and* F. verticillioides *at a concentration of 100 µg with positive control of nystatin. The zone of maximum inhibition was witnessed in the range of 7–14 mm against the fungal strains (166). Methanolic extract of the leaves repressed the development of *Enterococcus *species and* S. aureus* at a dose of 1–2 mg/ml (167). Acetone, methanol, ethanol, and chloroform extracts of the plant roots demonstrated noteworthy microbicidal potential against *S. aureus* and *K. pneumonia* (168). Leaf, stem, and root extracts of the plant were studied against *F. crown* for antifungal potential at a concentration of 100 mg/100 ml of solvent. Aqueous and organic solvent extracts of the aforesaid parts of the plant revealed supreme inhibition at a range of 5–45 mm with the positive control, dimethyl sulphoxide (DMSO) (169). Tables 4 and 5 summarize the bactericidal and fungicidal potential of the plant.

***Antidote activity***

Snake venom consists of myo, neuro, cyto, and enzymatic-toxins (155). Recently, antidote properties of *W. somnifera* are reported against toxicity induced by arsenic (115). Hyaluronidases enzyme present in snake venom helped the dispersal of toxins in the extracellular matrix of the tissues in victims. A glycoprotein extracted from *W. somnifera* acted as a hyaluronidase inhibitor against the venom of *Naja naja *(cobra) and *Daboia russelii *(viper) when studied using the zymogram assay. The extracts also inhibited the activity of phospholipase A-2 (an enzymatic toxin) present in cobra venom (156,170). In another research, the aqueous extract of *W. somnifera* (whole plant) neutralized the PLA-2 induced toxic effects from the venom of *Naja naja *(171).

***Pesticidal and larvicidal activities***

Aqueous, methanol and *n*-hexane extracts of roots and shoots (5, 10, 15, and 20% weight and volume) were evaluated against *Phalaris minor* in crops. Results showed that aqueous sprouts and root extracts exhibited significant herbicidal activity against *P. minor* compared with other solvent extracts (116). Methanol, aqueous, and n-hexane extracts of roots and shoots exhibited significant herbicidal activity. It was proven that foliar spray and soil applications of aqueous and methanolic extracts showed a reduction in germination and seedlings of *Parthenium hysterophorus *(133). 

Acetonic extract of the plant leaves was investigated against instar larvae and pupae of *Spodoptera litura*. Studies showed that pesticidal activity happened by causing toxicity, reduced transformation of larval-pupal, and adult intermediates. Thus, *W. somnifera* can be used as an insect growth regulator, which caused disruption at molting and metamorphosis stage of growth (147). Aqueous extract of leaves, stem, and roots of the plants was investigated for herbicidal activity against *Ageratum coenyzoides, Chenopodium album, *and *Achyranthus aspera.* It was established that aqueous extract of leaves is a more potent herbicid than stem and root extracts (154).

*W. somnifera* along with other plants such as *Clerodendrum inerme,* *Gliricidia sepia*, *Cassia tora*, and *Eupatorium odoratum *were extracted with ethanol to evaluate insecticidal potential against *Sitophilus oryzae. *It was observed that the mortality rate of insects was increased on increasing concentration (2.5 and 5%) of ethanolic extract during the 45 day study (158). The whole *W. somnifera* plant was extracted with petroleum ether to assess larvicidal action against *C. quinquefasciatus*, *A. stephensi, *and *A. aegypti. *It was discovered that the extract killed the larvae with an LC_50_ value of less than 100 ppm (159). Aqueous extract of roots and shoots was investigated for the herbicidal potential against *Rumex dentatus*. The application of extract reduced the length of roots and shoots significantly. The excerpt also decreased the biomass of seedlings of *R. dentatus* (171).

***Anti-inflammatory/anti-arthritic/analgesic activities***

Inflammatory diseases are associated with various types of rheumatic disorders such as rheumatic fever, ankylosing spondylitis rheumatoid arthritis, systemic lupus erythematosus polyarthritis nodosa, and osteoarthritis. Anti-inflammatory phytomedicines are effective inhibitors of cyclooxygenase mediated arachidonic acid metabolism responsible for producing prostaglandins that induce erythema, pain, and edema in various animal models. Purified phyto-drugs can act as a template for the synthesis of new anti-inflammatory drugs with low toxicity, cost, and high therapeutic values (5). 

Withaferin A suppressed the arthritic syndrome without any side effects when administered at 12–25 mg/kg of body weight to albino rats having adjuvant-induced arthritis. However, the administration of withaferin A to animals with arthritic syndrome increased body weight which was opposite to hydrocortisone sodium succinate. Results showed that withaferin A is more potent than hydrocortisone sodium succinate (43). Alcoholic extract of the plant leaves depicted significant anti-inflammatory activity by inhibiting tumor necrosis induced activation of Iκβ kinase responsible for activation of NFκβ. It was disclosed that the extract contained withaferin A, the only withanolide which can inhibit Iκβ kinase activation (88). 

Chloroform and aqueous extracts of leaves were investigated for inhibition of cell proliferation by inducing cell cycle seizure at Go/G1 and G2/M phase and limiting the expression of regulatory proteins. Both extracts controlled the expression of tumor necrosis factor (TNF-α), interleukin protein (IL-1β, IL-6) with a reduction in the production of reactive nitrogen and oxygen species by down-regulating the NFκβ and activator protein 1. Extracts also restricted the migration of active microglia with the down-regulatory expression of metalloproteinase. The extracts were also investigated effective in suppressing neuroinflammation and treatment of neurodegenerative disorders (138). Alcoholic extract of the roots displayed anti-inflammatory activity by inhibiting edema when administered at a dose of 12–25 g/kg of body weight in albino rats. It was concluded that a single dose of withaferin A showed prominent anti-inflammatory activity even after 4 hr of administration (139). Extracts of the plant leaves with alcohol produced anti-inflammatory and hepatoprotective effects. It was found that extract (1.0 g/kg of body weight) was as effective as phenylbutazone (50 mg/kg of body weight) and hydrocortisone (10 mg/kg of body weight) (141). Fresh leaves of the plant were extracted with methanol and water to investigate the anti-inflammatory activity in adult zebrafish of equal size and weight using reverse transcription-polymerase chain reaction. The extracts inhibited TNFα channel in zebrafish due to phenolic acids and flavonoids (142).

Anti-arthritic and anti-inflammatory effects of the plant were studied in adjuvant arthritic rats at a concentration of 1000 mg/kg and results were compared with indomethacin. The promising anti-arthritic potential was examined by stabilizing lysosomal enzyme activity (143). Administration of root powder at a dose of 600 mg/kg of body weight to collagen-induced arthritic rats considerably suppressed the severity of arthritis with improvement in functional recovery of motor activity and radiological score (145).

Withaferin A extracted and purified from *W. somnifera* exhibited anti-inflammatory activity by targeting cysteine-179 IKKβ and inhibiting NFκβ activation. The results were comparable to that of the standard drug, hydrocortisone sodium succinate (172). Various methanolic fractions of whole plant extract retained the anti-inflammatory activity comparable to hydrocortisone sodium succinate (5 mg/kg of body weight) due to the presence of withanolides (173). The anti-inflammatory potential of the plant might also be due to lymphocyte proliferation and delayed hypersensitivity depending upon the inflammation model, such as adjuvant-induced arthritis, carrageenan-induced, and cotton pellet granuloma inflammation model (174, 175). 

The anti-inflammatory potential of whole plant alcoholic extract was explored and found to be more significant than the standard hydrocortisone drug, owing to the presence of steroids (176). In another study, rats were injected with 3.5% formalin in hind leg footpads which reduced glucose absorption in the jejunum. The plant extracts maintained the absorption of glucose at normal levels while producing anti-inflammatory effects (177). A group studied the effect of aqueous root extract of *W. somnifera*, and glucosamine sulfate on nitric oxide-induced cartilage damage in chronic osteoarthritis patients. It was revealed that the extract significantly lowered nitric oxide release in patients (178).

Another study (1984) showed that the plant caused a dose-dependent suppression of macroglobulin in the serum of rats which was an indicator of anti-inflammatory activity (179). Administration of root powder at a dose of 1,000 mg/kg of body weight when orally administered to Wistar rats reduced the glycosaminoglycan content (92%) in granuloma tissues. The results are much better than the standard drug, hydrocortisone (43.6%) (140, 180). 

Similarly, hydro-alcoholic plant extract possessed significant anti-inflammatory activity due to withanolides and alkaloids against *in vitro *protein denaturation (181). Another study (2011) supported the anti-inflammatory effect of methanolic and chloroform extracts of the plant by analyzing cholinesterase and lipoxygenase inhibition activity at IC_50_ value of 69–111 and 76–132 µg/ml, respectively. Moreover, chloroform extract showed more significant anti-inflammatory activity thus supporting the folk medicinal use of *W. somnifera* by traditional healers (182).

The whole plant was extracted with ethanol (80%) and administered intraperitoneally to rats in which paw edema was induced by carrageenan using acetylsalicylic acid as a standard drug. The plant extract demonstrated significant anti-inflammatory potential at LD_50_ of 10 ml/kg of body weight. The anti-inflammatory activity of *W. somnifera* was found higher than *M. communis, M. chamomilla, A. graveolens*, and* A. santolina* (183). In another study, the extract of plant delayed the analgesic effect induced by morphine. It also suppressed the rebound hyperalgesia induced by morphine in the tail-flick test probably (184). 

The ethanolic root extract of *W. somnifera* (12–25 mg/kg of body weight) was orally administered to albino rats (185). The effects of whole plant extracts (100 or 200 mg/kg of body weight) were evaluated against the pentylenetetrazol seizure threshold in mice. It was revealed that plant extract increased the PTZ seizure threshold in a dose-dependent manner (186). Anti-inflammatory activity of aqueous root extract of *W. somnifera* was studied by evaluation of TNFα, Inter Leukin (IL) IL-1β, IL-6, and IL-10 in collagen-induced arthritis in rats. Oral administration of aqueous root extract of *W. somnifera* (300 mg/kg) attenuated the transcription factors of arthritis in rats by lowering the reactive oxygen species and metaloproteinase-8 level to normal in collagen-induced arthritis bearing rats (187). 

The methanolic root extract of *W. somnifera* showed a protective effect against morphine-induced analgesic tolerance by the spine density reduction mechanism in rats. The biological mechanism involves activation of peroxisome proliferator-activated receptor γ which produced pro-longer protection against morphine-induced analgesic tolerance. The methanolic root extract of *W. somnifera* (100 mg/kg of body weight) was administered in reference with morphine (10 mg/kg of body weight) and peroxisome proliferator-activated receptor γ antagonist GW-9662 (5 and 10 µM) to male Sprague rats. Results showed that peroxisome proliferator-activated receptor γ antagonist shows functional capability for attenuation of prolonged morphine analgesic effect along with reduced tolerance after repeated administration of methanolic root extract of *W. somnifera*. Moreover, peroxisome proliferator-activated receptor γ antagonist (5 and 10 µM) and *W. somnifera* (1.00 mg/ml of methanolic root extract) were administered to cell culture line SH-SY5Y for evaluation of the protective effect on µ-opioid and peroxisome proliferator-activated receptor γ receptor. Cell culture analysis reveals that blocking of peroxisome proliferator-activated receptor γ receptor by GW-9662 helps in the down-regulation of µ-opioid m-RNA which in turn enhances the availability of the µ-opioid receptor for analgesic effect (188).

The root extract of *W. somnifera* was investigated for analgesic effects in a plantar incision, mechanical withdrawal threshold, and spared nerve injury models in rats by quantifying the interleukin and interferon biomarkers in the dorsal root ganglia of rats by ELISA cytokine assay. Results showed that significant increase of mechanical withdrawal threshold, spared nerve injury-induced hyper-analgesia, and cytokine levels were observed in a dose-dependent manner after 6 and 24 hr administration of *W. somnifera* root extract at doses of 100 and 300 mg/kg of body weight. Withaferin A, the main active compound of *W. somnifera* roots, seemed to be responsible for chemochine receptor family 2, which shows analgesic effect in the post-operative and neuropathic treatment of rats (189).

Analgesic effect of *W. somnifera* was attributed to the capability for reducing the level of serotonin, which was majorly responsible for the pain in the body. Ethanol (0.05855 g/ml), butanol (0.05135 g/ml), xylene (0.0628 g/ml), and methanolic (0.0541 g/ml) root extracts of *W. somnifera* were administered to albino mice at 2 hr to 12 hr time intervals. Serotonin concentration was observed by UV-visible spectroscopy which clarifies the reduction of serotonin level. Results showed that ethanol-based extract showed more significant inhibition of serotonin production followed by methanol-based extract, while the other two didn’t show significant results for the reduction of serotonin (190).

***Anti-tumor/cytotoxic activities***

Later on, in 2002, Davis and Kuttan noted the enhanced proliferation rate of lymphocyte, thymocyte, and bone marrow after administration of plant powder at a dose rate of 20 mg/dose/animal to splenocyte which was pre-treated with PHA and Con A mitogens (81). Withanolides isolated from *W. somnifera* inhibited the growth of cancerous cells in the central nervous system, lungs, breasts, and colon cell lines. It was investigated that withaferin A significantly reduced the growth of breast and colon cancer cell lines more effectively than famous anticancer drug doxorubicin (85). Blocking of NFκβ activation sites may involve the inducible or constitutive mechanisms of suppression which resulted in the elevation of apoptosis, inhibition of invasion, and osteoclastogenesis. Withanolide D isolated from leaves of *W. somnifera* showed exceptional antileukemic activity. The antileukemic activity of withanolide D was mediated by ceramide accumulation after activation of *N*-SMase2 which in turn enhanced the apoptotic activity of neoplastic cells (90). 

Antitumor activity of the ethanolic root extract of *W. somnifera *was evaluated against Dalton’s ascitic lymphoma in Swiss albino rats. It was discovered that extract decreased tumor size, weight, and the number of cancer cells significantly (125). Anti-proliferative activity in reference to structure-activity relationship for withanolides confirmed the presence of 2,1-oxo-functionality in ring A, 5, and 6β-epoxy or 5α-chloro-6β hydroxy groups in ring B against the human head, breast, and neck squamous carcinomas cell lines (146). *In vitro *cytotoxic evaluation of 50% ethanolic extract of root, stem, and leaves against different human cancer cell lines, e.g., prostate, lungs, colon, and neuroblastoma was appraised. It was revealed that ethanolic leaf extract exhibited morte potent antitumor activity against prostate and colon cancer than roots and stem extracts (153). The mechanism involved behind the antitumor activity is retardation of cyclooxygenase enzymes, the proliferation of tumor cells, and lipid peroxidation by inhibiting the activation of nuclear factor-κβ (NF-κβ) at the genetic level (187). 

Withaferin A exhibited *in vivo* anti-angiogenic activity by inhibiting the transcription factors for vascular endothelial cell growth at very low concentrations (191). Withaferin A, physagulin D, 4-(1-hydroxy-2, 2-dimethylcyclpropanone) 2,3-dihydrowithaferin A, sitoindoside IX, physagulin D (1→6)-β--glucopyranosyl-(1→4)-β -glucopyranoside, 2,3-dihydrowithaferin A, 24,25-dihydro-27-desoxywithaferin A, 27-*O*-β--glucopyranosylphysagulin D, 27-*O*-β--glucopyranosylviscosalactone B, 4,16-dihydroxy-5β, 6β-epoxyphysagulin D, withanoside IV, and viscosalactone B isolated from alcoholic leaf extract were assessed for antiproliferative activity on NCI-H460 (lungs), HCT-116 (colon), MCF-7 (breast), and SF-268 (central nervous system) human cell lines. Withaferin A with its derivatives viscosalactone B, and 27-*O*-glucoside derivatives exhibited significant antiproliferative activity and the IC_50_ values ranged from 0.01–11.6, 0.05–0.47, and 2.9–17.3 μg/ml, respectively (80). The pretreatment of Wistar rats before exposure to UV radiations with withanolide, 1-oxo-5*β*, 6*β*-epoxy-witha-2-enolide (20 mg/kg) isolated from the roots of the plant prevented the reoccurrence of skin cancer (192). 

The protective effect of withaferin A on the integrity of red blood cells was evaluated in dimethylbenzanthracene induced oral carcinogenesis by measuring glycol-conjugates, red blood cell osmotic fragility, and membrane-bounded enzymatic activity. It was disclosed that oral administration of withaferin-A (20 mg/kg of body weight) for 14 weeks barred tumor incidence in the golden hamster completely (193).

Various extracts (aqueous, alcoholic) of different parts of the plant revealed anti-carcinogenic potential with decrease in the activity of NFκβ which resulted in the suppression of intercellular tumor necrosis in cancerous cell lines. The extract also reduced tumor size and count. Mice were fed the plant before and during exposure to skin cancer inducer, 7,12-dimethylbenzanthracene, for evaluation of the chemopreventive effect. A significant decrease in prevalence and count of skin lesions was observed with no change in enzymatic level and lipid profile (194, 195).

The plant was also evaluated for the anti-carcinogenic activity against urethane-induced lung cancer in adult male albino mice. Results showed that the simultaneous intake of the powdered plant (200 mg/kg, daily) and urethane (125 mg/kg, biweekly) for seven months significantly lessened the incidence of tumors (196). Antiproliferative activity of the plant was assessed against human laryngeal carcinoma (Hep2) cells using microculture tetrazolium assay. Retardation of cell (Hep2) viability was observed due to cyclic arrest and agglomeration of hypoploid cells (197). A significant increase in life span and a decrease in tumor weight with cancer cell numbers were observed in mice after oral administration of plant powder to mice (198). 

Anti-cancerous protein fraction was extracted from *W. somnifera* roots, which showed activity against the human MDA-MB-231 breast cancer cell line. The action of protein fraction was mediated by reactive oxygen species dependent mitochondria-mediated apoptosis mechanism in the breast cancer cell line. The shade-dried roots (100 g) of *W. somnifera* were suspended in 400 ml of 0.1M trisphosphate buffer at 4 °C with continuous stirring at 12000 rpm for 20 min. The extracted protein was purified and concentrated at 3.0 mg/ml concentration, and anti-cancerous activity was studied by MTT assay against MDA-MB-231 cell line showed that the arrest of the G2 phase was observed in dose-dependent manner results the stalling of mitotic progress (199). 

The ethanolic root extract of *W. somnifera* was applied at a dose of 0.05–0.4 mg/ml to leukemic THP-1 and peripheral blood mononuclear cells for 24 to 72 hr. Results showed that after 24 hr treatment, increase in leukemic THP-1 and PMBC viability was observed. However, peripheral blood mononuclear cell viability remains increased with decrease in leukemic THP-1 and inhibition of cell growth to 50% for HT-29, HCT-15, SW620, 502, 713, Colo-205, A549, HOP-62, and Hep-G2 cell lines at 30 µg/ml of the extract after 72 hr (200).

***Anti-oxidant and hepatoprotective activities***

Lipid peroxidation activity of aqueous suspension of roots was investigated by administration to mice and rabbits at a dose of 100 mg/kg after 6 hr intervals. The concentration of lipid peroxide was decreased in *K. pneumoniae* and *S. aureus* which advocated the prophylactic activity against stress induce lipid peroxidation (126). It was suggested that the anti-oxidant potential of withanolides might be due to the hydroxylated long chain of the carbon-bearing acyl group. Other compounds such as sitoindosides VII-X and withaferin A were investigated as potent initiators for free-radical scavenging enzymes, catalase, glutathione peroxidase, and superoxide dismutase in the striatum and frontal cortex of rat’s brain (160). 

Another study revealed the protective effect of aqueous extract of the whole plant (500–1000 mg/kg of body weight) in paracetamol-induced hepatotoxicity. The extract reversed the effects of hepatotoxicity by lowering the concentration of liver marker enzyme, bilirubin, with improvement in protein contents (161). Alkaloids (withanamides A-I) extracted and purified from *W. somnifera* were assessed for anti-oxidant activity using a large unilamellar vesicle model. It was disclosed that withanamides (A-I) isolated from the plant fruits retarded lipid peroxidation significantly at a concentration of 0.5-1 μg/ml. It was also noticed that withanoside V displayed prominent free radical scavenging activity at 10 μg/ml concentration (98, 201). Elevation in the enzymes showed increased anti-oxidant potential with a protective effect on neural tissues (202-205). 

Aqueous extract of the roots was tested for the anti-oxidant effect in male albino rats against cypermethrin induced oxidation. Extract, when administered at a dose of 5 ml (10% root’s extract) for 60 days to male albino rats, showed the complete restoration of all biochemical and hematological parameters (206). Co-administration of methanolic extract of roots of *W. somnifera, *leaves of *Ocimum sanctum*, and rhizome of *Zingiber officinale *reduced tenuous physical exercise and swimming-induced oxidative damage in Wistar rats. The stresses significantly elevated the number of free radicals which lowered the activity of catalase, superoxide dismutase, and glutathione-S-transferase in secondary sex organs. Co-administration of the aforesaid extracts at a dose of 0.5 ml/100 g of body weight helped to increase anti-oxidant activity with regaining a reasonable level of enzymes (55,207).

Glycowithanolides (sitoindosides VII-X, withaferin A) isolated and purified from roots of *W. somnifera* were administered to rats at doses of 10, 20, and 50 mg/kg of body weight for 10 days. The extracts reduced iron-induced hepatotoxicity due to the anti-oxidant activity of glycowithanolides (208). The powder of roots affected the circulatory level of urea, ammonia, lipid peroxidation products (hydroperoxides, thiobarbituric acid reactive substances), and liver marker enzymes (alanine transaminase, aspartate transaminase, and alkaline phosphatase) showing hepatoprotective potential.  The plant elevated the level of hepatic protection by affecting the concentration of liver markers and lipid peroxidation products in experimental hyperammonemia. The hepatoprotective activity might be mediated by the controlling mechanism of alkaloids, withanolides, flavonoids, urea, and urea related compounds (209). Lesions induced by carbendazim in the liver and kidney were completely cured using the powder of plant roots for 48 days (210). Methanolic extract of the plant exhibited significant free radical scavenging potential and protected DNA damage induced by hydrogen peroxide (211). 

***Immunomodulatory activity and hematopoiesis***

An increase in the production of nitric oxide owing to activation of nitric oxide synthase in mouse macrophages was observed after the administration of methanolic extract of the plant roots (1–256 μg/ml) (94). Evaluation of the immunomodulatory effect of purified sitoindoside IX and X from the plant on the central nervous system as anti-stress agents was studied at a dose rate of 100–400 g/mouse. It was concluded that significant activation and mobilization of peritoneal macrophages and phagocytosis enhanced the lysosomal enzymes secreted by the macrophages. It was further confirmed that sitoindosides reduced the deficits in the cerebral function of the geriatric population (108). *In vitro* and *in vivo* immunomodulatory effect of plant root powder was evaluated and the potent inhibitory effect on mitogen-induced lymphocyte proliferation with delayed hypersensitivity in mice was explored (174). Immunomodulatory effects of alcoholic extract of the plant roots were assessed in cyclophosphamide, azathioprine, or prednisolone myelosuppression models in mice. The extract enhanced the number of blood cells, bone marrow cellularity, and α-esterase positive cell number count (127, 211, 212).

It was discovered recently that the mechanism of immunomodulation involved phytochemicals such as 2,3-dihydrowithaferin -A-3-β-*O*-sulfate, daucosterol, withasomniferol-A, withaferin-A, and β-sitosterol, which regulated multiple immunity pathways via bioactive-targets and protein-protein interactions (213). In a similar study, it was found that a mixture of sitoindosides IX, X, glycol, and anolides isolated from the plant statistically enhanced the immunomodulatory effect by activation of macrophages and lysosomal enzymes (214). The aqueous whole plant extract, when administered to albino rats, showed a significant increase in the production of antibodies which reduced mortality with improved immune response (215). Efficacy of various antileishmanial drugs (miltefosine, paromomycin, and amphotericin B) has been enhanced when used in combination with root extract of the plant containing withanolides in *Leishmania donovani* infected hamsters (216). 

***Neurotic regeneration activity***

The neurodegenerative disorder is actually the selective dysfunctioning or sometimes death of neural cells in the central nervous system or regeneration of synaptic, neuronal, and neurotic cells (5, 217-222). The plant extract was fed to a group of mice for three weeks. It was declared that the extracts showed neuroprotective and neuronal growth effects by reversing all neurodegenerative processes (13). The methanolic root extract of the plant exhibited prominent neurite regeneration even at a dose of 1.0 mM on a human neuroblastoma SH-SY5Y cell line (80). Different derivatives of withanolides such as (20S,22R)-3*,*6α-epoxy-4*,*5β,27-trihydroxy-1-oxowitha-24-enolide, 27-O-β-D-glucopyranosylpubesenolide 3-O-β-D-glucopyranosyl (1→6)-β-D-glucopyranoside, 27-O-β-D-glucopyranosyl (1→6)-β-D-glucopyranosylpubesenolide 3-O-β-D-glucopyranosyl (1→6)-β-Dglucopyranoside, 27-O-β-D-glucopyranosylpubesenolide3-O-β-D-glucopyranoside, and (20R,22R)-1α,3β,20,27-tetrahydroxywitha-5,24-dienolide 3-O-β-Dglucopyranoside) isolated from methanolic extract of roots produced positive neurite outgrowth effects in rats (86). Sominone, a class of withanoside IV, was the major metabolite responsible for significant axonal and dendritic regeneration with synaptic reconstruction in Alzheimer’s disease (92). 

Sitoindosides (VII–X) and withaferin extracted from roots were investigated on brain cholinergic, gamma-aminobutyric acid-induced allergic and glutamatergic receptors in rats. Results showed a slight increase in acetylcholinesterase activity in lateral septum with relative lowering in the vertical diagonal band of lateral septum and globus pallidus (128). The root extract of the plant reversed the effects of scopolamine-induced disruption, retention, and attenuation in amnesia (129). It was revealed that withanolide A at a dose of 10 mmol/kg of body weight reconstructed severely damaged neurons in mice (11, 203). 

Methanolic extract of *W. somnifera* roots was administered to male Sprague mice at a dose rate of 200–400 µg/ml to access the morphine and ethanol ability to stimulate the ventral tegmental area dopaminergic neurons and transmission of dopamine. Results showed that morphine and ethanol significantly reduce the neural response of the ventral tegmental area and dopamine in nucleus accumbens by GABA_A _mechanism. Oral administration of *W. somnifera* extract at 75 mg/kg of body weight helps in the reduction of morphine and ethanol elicited increase in nucleus accumbens of rats (221). The mechanism involved for the neuroprotective potential was mediated by the retardation of nitric oxide production which was responsible for the neurodegeneration activity of the brain in mice (223).

The plant significantly suppressed the haloperidol-induced catalepsy with the provision of hope for the treatment of Parkinson’s disease (224). Glycowithanolides, isolated and purified from methanolic plant extract were administered in combination with haloperidol for four weeks, inhibited neuroleptic tardive dyskinesia (225). 

Post-traumatic stress disorder was treated with administration of root powder of *W. somnifera* at a dose of 500 mg/kg/day to rats. Radial arm water maze and enzymatic markers assays were used to access spatial memory, learning, and oxidative stress biological markers in rats. Results showed that the administration of the root powder of *W. somnifera* prevented memory impairment in rats after post-traumatic stress disorder by affecting anti-oxidant mechanisms in the hippocampus part of the brain in rats. The study led to concluding that *W. somnifera* can be used for the treatment of neurodegenerative diseases induced by post-traumatic stress disorder (226).

Cognitive dysfunction along with spatial learning defects induced by bisphenol A was improved by administration of ethanolic root extract of *W. somnifera* at a dose of 100 mg/kg of body weight per day to white albino mice. Neurodegeneration and spatial learning defects were measured by Y maze and Morris water maze assays. Results showed that impairment caused by bisphenol A was reduced by the administration of *W. somnifera* extract after recovering the NMDA receptor in the hippocampus region of albino mice (227).

***Adaptogenic activity***

5,6β-epoxy-1-oxo-witha-2-ene-27-ethoxy-olide purified from plant roots was studied for the anti-stress, lactate dehydrogenase, serum creatine phosphokinase, serum corticosterone level, and serum lipid peroxidation (55). The standardized root extract of *W. somnifera* was investigated against chronic stress, hyperglycemia, plasma corticosterone, and male sexual dysfunction in male Wistar rats. The results showed that the root extract (concentrations of 25 and 50 mg/kg) exhibits significant anti-stress and adaptogenic activities in stress-induced immunological perturbations of mice (87). 

Adaptogenic herbs were primarily used to strengthen the immune response of the human body, which controlled the level of stress hormone in human blood. Whole plant extract when administered in stressed animals, reduces urinary tribulin level which stimulated physical and mental health, augmented resistance, and increased longevity. With the adaptogenic capability, the plant helped in the reduction of muscle proteins with the provision of perfect natural anabolic aid for athletes (207, 208, 228). The significant anti-stress activity was observed for the defatted alcoholic extract of plant seeds when administered intraperitoneally at a dose of 100 mg/kg to mice (229).

Sitoindosides VII and VIII (50–100 mg/kg) showed a significant anti-stress effect in forced swimming induced immobility and gastric ulcers in mice (230). The alcoholic whole plant extract when orally administered (100 mg/kg of body weight) twice a day reduced stress-induced elevation in blood urea level, blood lactic acid, and adrenal hypertrophy in rats (231). The extract improved the swimming duration of mice with restoration of plasma cortisol, blood glucose, and triglyceride levels (232). The role of the plant for synergistic activation of the differential gamma-aminobutyric acid receptor as a potential pathway for the adaptogenic and neurological disorders (anxiety, nervous exhaustion, insomnia, etc.) in mice was investigated and found to be prominent (233).

***Obsessive-compulsive disorder***

Various extracts of the plant had been used to alleviate mood in patients suffering from behavioral disturbances. Aqueous extract of plant roots was orally administered at doses of 50, 100, and 200 mg/kg to mice in electroconvulsive shock treatment. It was noticed that the extract improved the retention in step-down paradigm and scopolamine-induced disruption in mice during electroconvulsive shock treatment (129). Alzheimer’s disease was the result of ibotenic acid-induced lesioning in magnocellular basal nuclei, which produced cognitive scarcity. The equimolar dose of sitoindosides (VII-X) and withaferin A at a concentration of 20–50 mg/kg considerably reduced the effects of ibotenic acid (159). Significant reduction in the frequency of micronucleated polychromatic erythrocytes and chromosomal aberrations was observed in the golden hamster after treatment with withaferin A extracted from the plant (193).

Glycowithanolides extracted from the plant roots were assessed for anxiolytic and antidepressant activities at doses of 20 and 50 mg/kg for 5 days in rats. The results of the extract were comparable to those of the standard drug, benzodiazepine lorazepam (208, 234). The plant also increased the spent time and entries in open arms of elevated plus maze test and showed anxiolytic activity in a dose-dependent manner. The methanolic and aqueous plant extracts gradually lowered marble-burying behavior without affecting motor activity in reference to fluoxetine, parachlorophenylalanine, and ritanserin. The neuroprotective properties of *W. somnifera* root extracts (50 mg/kg of body weight) in mice were evaluated for the treatment of the disorder in mice using a marble covering model. The extract depicted a lessened marble covering activity as compared to the standard drugs such as fluoxetine, parachlorophenylalanine, and ritanserin (235). In another investigation, mice treated with daily oral doses of plant root extracts (10, 20, or 40 mg/kg) showed enhanced marble burying tests in a dose dependant fashion. The extract was also investigated effective against foot shock stress-induced hyperthermia. Thus the plant is beneficial for treating antidepressant and anxiolytic activities (236). 

***Cardioprotective activity***

Root powder of the plant when orally administered to hypercholesteremic rats reduced total lipid cholesterol and triglycerides contents in subject animals. However, a significant increase in HDL cholesterol level, liver bile, and 3-hydroxy-3-methylglutyral-Coenzyme A reductase activity was also observed (18). Later on, it was noticed that tuber powder of *W. somnifera* (50 mg/kg of body weight) administered to albino rats showed cardioprotective and anti-oxidant activities in isoproterenol-induced myocardial infarctions (163).

The alkaloids isolated from roots of *W. somnifera* plant exhibited prolonged hypotensive, respiratory stimulant, and bradycardiac actions in dogs (237). The cardioprotective effect of hydro-alcoholic extract of *W. somnifera* at doses of 25, 50, and 100 mg/kg was investigated in isoprenaline (isoproterenol) induced myocardial necrosis using rats as model animals. A significant cardioprotective effect with continuous restoration of the hemodynamic parameter was observed. Various extracts of the plant had been used to increase the beating ratio of the heart by lowering the lipid peroxidation level (238, 239). 

Another investigation was focused on the anti-apoptotic activity of the plant in Wistar rats at a dose of 50 mg/kg. The mechanism behind the process involved the up-regulation of BCl-2 (anti-apoptotic protein) and down-regulation of Bax, pro-apoptotic protein (240). The cardioprotective effect of the plant extracts at a concentration of 40 mg/kg of body weight in an isolated rat heart model was observed. It was discovered that the extract had significant cardioprotective ability in ischemia and reperfusion injuries with reduced infarct size (241). Various pharmacological attributes of the plant are summarized in Table 6.

***Toxicity***

Significant consideration was given to the diligent evaluation of cytotoxicity induced by phytochemicals extracted from *W. somnifera *while investigating the therapeutic properties (242). Acute and subchronic toxic effects caused by the plant in Wistar rats were assessed by oral administration of whole plant extract at a dose of 500, 1000, and 2000 mg/kg body weight/day for 28 days. Histopathological parameters, serum analysis, hematology, and body weights were assessed at the end of the study. The results showed that acute toxicity was observed at a dose of 2000 mg/kg of body weight in Wistar rats (243, 244). Fenvalerate-induced neurotoxicity led to developing convulsion, weight loss, tremors, and paralysis in cockerels. *W. somnifera* root powder (200 mg/kg of feed), when administered to the cockerels, lowers the severity of fenvalerate-induced toxicity with progressive improvement in red blood cell count, total leucocyte count, and total erythrocyte count (245).

Acute and chronic toxicity evaluation of the hydro-alcoholic extract of roots was carried out by administering to female albino rats at a dose of 1000 mg/kg of body weight. Research showed the initial excitement followed by dullness, depression, reduced motor neuron activity, and decreased respiration (246). Another study concluded that LD_50_ for alcoholic root extract was 1,260 mg/kg in mice but no acute mortality was observed at 1,100 mg/kg. With a further increase in dose concentration at a rate of 100 mg/kg, there was a significant increase in the death rate (247).

***Miscellaneous***

*Aloe* *vera* based herbal formulation of ethyl acetate extract of *W. somnifera* fruits was prepared to treat alopecia in male Wistar rats. Results showed that good growth of hair follicles was observed due to the anti-oxidant property of the extract. The anti-oxidant activity made the extract suitable for direct use on the skin after testing it for erythema and edema up to 48 hr in rats (248).

Nicotine withdrawal usually induces anxiety with an increase in locomotor activity and sensitization which was reduced to the lowest possible value after treatment with *W. somnifera* extract at 100 mg/kg of body weight. Nicotine biologically induces anxiolysis which was effectively blocked by *W. somnifera* extract which reduces hyperactivity by positively affecting GABAgeric and serotonergic parameters which are anti-stress agents (249). 

Treatment of sputum smear-positive pulmonary tuberculosis was treated with the root extract of *W. somnifera* for 12 weeks. Blood profile, CD-4, CD-8, body weight, erythrocyte sedimentation rate, serum glutamic oxaloacetate transaminase, and serum glutamic pyruvic transaminase was studied. After 12 weeks of treatment significant increase in CD-4, CD-8, serum glutamic oxaloacetate transaminase, and serum glutamic pyruvic transaminase were observed, which gives positive effects, improves the immunological parameters of patients, and helps in the treatment of TB (250).

The ethanolic root extract of *W. somnifera* exhibited significant anti-hyperlipidemic effects in male rabbits when administered at a dose of 50 and 100 mg/kg of body weight along with a high cholesteric diet thrice a day. Administration of the extract led to conclude that no significant change in the lipid profile of male rabbits was observed even after the administration of a high cholesteric diet. Results showed that the property of root extract lowers the total cholesterol and triglycerides and 3-hydroxy-3-methylglutyral-Coenzyme A reductase, which might be attributed to the presence of polyphenols and flavonoids in root extract (251).

Methanol, dimethylsulfoxide, *n*-hexane, and acetone based extract of *W. somnifera* whole plant buffered with phosphate buffer saline to give 0.05 mg of plant extract/ml was administered to *Haemonchus contortus *for 6 hr. Results showed that the high mortality rate of *H. contortus *which was comparable to the Levamisole standard anthelmintic agent, which shows 100% motility in 4 hr at 0.05 mg/ml. In comparison to all extracts, the acetonic extract of *W. somnifera* exhibits 100% anthelmintic activity in 6 hr which is comparable to standard Levamisole (252). An enzyme, α-amylase inhibitor extracted from *W. somnifera,* was used to improve the quality of potato chips. Potato chips were treated with 220 ppm solution of *W. somnifera* plant extract for 30 min, which results in the reduction of polyphenol oxidase activities and sugar content up to 40 and 25%, respectively (253). Synthetic pyrethroids (cypermethrin and deltamethrin) were administered at doses of 750 and 40 mg/kg of body weight, respectively to Wistar male rats. Combined administration of cypermethrin and deltamethrin results in a sudden decrease in body weights of Wistar rats and the effect was improved by giving a methanolic suspension of *W. somnifera* (12.5 mg/kg of body weight) in 3% gum acacia solution (254). *In vitro* results revealed that the methanolic extract of plant roots enhanced the multiplication of lymphocytes. The extract also upgraded antibodies in dexamethasone-induced immunocompromised mice (255). 

**Clinical trials**

***Male infertility***

Stress has been reported as a principal causative agent for impotence in males. Pre-historic studies showed *W. somnifera* had been investigated for the anti-stress activities (5). It was revealed in a study that the plant reversed the effect of sodium arsenite administration on sperm counts and motility and also maintained the cellular integrity of testicular cells leading to its normal functioning (115). In an investigation, reduction in lipid peroxidation and protein carbonyl concentration with improvisation in sperm count, motility, and seminal plasma levels were observed in infertile males on treatment with a whole-plant extract which reduces (256-258). In another exploration, the effect of stress on male infertility by pre- and post-stress treatments was studied by administering root powder at a dose of 5 g/day for three months. The extract of medications improved semen quality and anti-oxidant levels in male individuals (259-261). In a pilot study, women were administered 300 mg of highly concentrated aqueous root extract twice a day for eight weeks. It was found that the extract improved the physical and sexual dysfunction of women (261). Above mentioned studies revealed the potential of the plant to cure sexual disorders. However, further studies are neede for development of drugs from the plant in the future. 

***Cardioprotective, anti-cancerous, bone and muscle-strengthening activities***

In a double-blind placebo-controlled study, the effect of administration of a herbo-mineral formulation containing the root powder of the plant (450 mg/capsule) on osteoarthritis was studied. Biological studies of 42 patients showed a lowering of osteoarthritis pain and disability scores. Placebo study of alcoholic whole plant extract was conducted in 42 patients suffering from severe osteoarthritis. Results showed a significant reduction in pain and disability scores with no change in the erythrocyte sedimentation rate mediated by cyclooxygenase inhibition pathway (144). Placebo study for the cardioprotective effect of *W. somnifera* was investigated in adult athletes by oral administration of 300 mg capsule bearing highly concentrated aqueous root extract twice a day for twelve weeks. Findings showed that cardiorespiratory activity was enhanced with an elevation of physical level exertion in healthy adults (262). 

Few studies in human beings are available to investigate the anti-cancerous and bone and muscle strengthening potential of the plant. In one such study, administration of root extract of *W. *somnifera (2.0 g tds) during three courses of chemotherapy of breast cancer-bearing fifty women lowered fatigue with improvement of quality of life (263). Two groups of 35 individuals each suffering from sarcopenia were fed 500 and 750 mg of root extract for three months to improve the strength and functioning of muscles (264). Root extract, when administered to males at 300 mg, twice a day for 8 weeks, increased the strength, size, and recovery of body muscles along with serum testosterone (265). The findings of these studies are encouraging and will help in the future to isolate the bioactives responsible for these activities in pure form. 

***Immunomodulatory, hypolipidemic, and anti-diabetic activities***

Obesity and diabetes are prevailing particularly in developing countries due to malnutrition. Synthetic drugs are used to decrease the effects of diseases with side effects. Thus, there is a need to explore plants for curing various ailments. Root powder of *W. somnifera* administered for 30 days in hypercholesterolemic and diabetes mellitus patients lowered blood glucose with an increase in the concentration of sodium, volume, and low-density lipoproteins in urine (132). Anti-aging properties of the plant were assessed by double-blind clinical trials in 101 healthy males (50–59 years of age). The individuals were fed 3 g daily dose of the plant for one year. The youthful male patients experienced improvement in hemoglobin, seated statue, red blood cell count, and hair melanin. Lowering in serum cholesterol with preservation of nail calcium was also observed (198). In one study, it was revealed that the administration of 6.0 ml root extract to five patients for 96 hr increased the immunomodulatory effect mediated by the regulation of CD4, CD3+T, and CD56+NK cells (266). Further studies are needed to isolate pure bioactives from the plant for the development of formulations with lesser side effects. 

***Obsessive-compulsive disorder***

The obsessive-compulsive disorder is a condition of chronic mental anxiety, which might be attributed to dysregulation of the serotonergic network of neurons in the brain (267, 268). It is an obstinate, impairing mental issue that is characterized by pain-inducing steady and obsessive thoughts. Despite various medical treatments, significant numbers of patients (40–60%) were not willing to be treated by pharmacological experts due to social stigma. *W. somnifera* root powder at a dose of 4.5 g twice a day was administered to the patients suffering from compulsive disorder for 30 days, and results measured on Yale-Brown obsessive-compulsive symptom checklist showed significant improvement in mental activities with lowering of obsessive and painful thoughts by directly affecting gamma-aminobutyric acid receptors. The mitigation of mental nervousness achieved by *W. somnifera* showed long term effects, which were observed in multiple follow-ups of the treated patients (269). In another clinical finding, the treatment of 30 affirmed obsessive-compulsive disorder patients was accomplished using root extract of the plant at a dose of 120 mg/kg of body weight for six weeks under the selective serotonin reuptake inhibitors treatment. The results revealed no adverse effect associated with plant root extract on mental health, but behavioral improvements along with reduction of obsessive thoughts were observed in treated patients (270). It could thus be concluded that the plant roots possess the phytochemicals for the treatment of an obsessive-compulsive disorder.

The root powder of the plant was orally administered to Wistar Albino rats at a dose of 10 mg/kg body weight to investigate response against cold swimming stress. After the administration of the powder for seven days, animals were sacrificed on the 8^th^ day after exposure to cold water swimming stress. The study revealed that the plant root powder increased plasma corticosterone level, phagocytic index, and avidity index when rats were exposed to cold water swimming test (271). 

The anti-anxiety effect of ethanolic extract at a dose of 1000 mg/individual was assessed in the control group as prescribed in placebo tablet. Patients were assessed according to the Hamilton Anxiety Scale, systematic assessment for treatment-emergent effects, and the Global Rating Scale for weeks 2 and 6. Results tended to favor the usage of the plant in lowering the anxiety level with the lowest possible adverse effects (272). The anti-stress effect of roots and leaves was evaluated at doses of 125 mg once daily, 125 mg twice daily, and 250 mg twice daily in three different groups of patients for 60 days. The extracts lessened the anxiety and stress significantly in a dose-dependent manner. It also increased serum concentrations, fasting blood glucose levels, and lipid profile (273). A significant decrease in the Beck Anxiety Inventory score was observed with no adverse effects in 81 participants administered with withanolides at a dose of 300 mg, twice a day (274).

In another study, the anti-stress activity of the plant was appraised in 64 patients for 60 days. Participants were administered with 600 mg of highly concentrated root extract twice a day. There was noted a significant difference in cortisol level of bloodstream with non-significant side effects (275). In another investigation, W. somnifera revealed significant improvement against anxiety disorder in patients. The primary impact observed was on the “anxious mood” in participants receiving treatment with W. somnifera (276). 

**Table 1:** Important withanolides with chemical names, structures, and biological activities

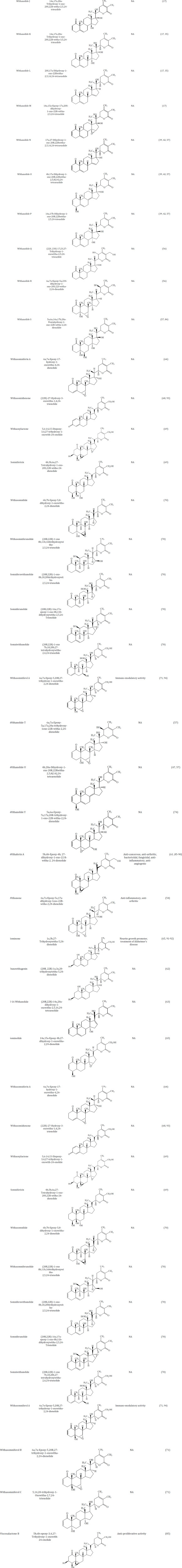

**Table 2:** Summarized phytochemistry of various parts of *Withania* *somnifera*

Plant part used	Phytochemicals isolated	Nature of extract	References
Leaves	Anaferine (bis (2-piperidylmethyl) ketone), tropine, isopelletierine, 3*α*-tigloyloxtropine, pseudotropine, cuscohygrine, 3-tropyltigloate, anahygrine, hygrine, *dl*-isopelletierine, mesoanaferine, somniferine, choline, hentriacontane, withanine; withananine, withasomnine, visamine, ashwagandhine, and pseudowithanine	Methanolic	[35]
	Withanolide D, N, O, P	Alcoholic	[39,42,57]
	Withanolides	Methanolic	[37-39]
	Withanolides G–M	Alcoholic	[17]
	Withanolide F,T, and U	Alcoholic	[57]
	Withanoside IV, physagulin, and withanoside VI	Butanol	[96]
Leaves and roots	27-hydroxy withanone, 17-hydroxy withaferin A, 17-hydroxy-27-deoxy withaferin A, withaferin A, withanolide D, 27-hydroxy withanolide B, withanolide A, withanone, and 27-deoxywithaferin A	Methanolic	[77]
Roots	Withasomnine	Alcoholic	[33]
	Withanolide A	Alcoholic	[51]
	Pseudotropine, isopelletierine, 3*α*-tigloyloxtropine tropine, *dl*-isopelletierine-3-tropyltigloate, cuscohygrine, anaferine, hygrine, anahygrine, somniferine, mesoanaferine, choline, withanine, visamine, withananine, hentriacontane, withasomnine, along with pyrazole derivatives pseudowithanine and ashwagandhine	Methanolic	[53]
	Withasomniferol A, B, and C	Benzene, ethyl acetate	[71]
	*β*-sitosterol and *d*-glycoside	Petroleum ether, acetone	[75]
	Withanoside IV and withanoside VI	Butanol	[97]
	Ashwagandhanolide	Methanolic	[100]
	Withanosides I, II, III, IV, V, VI, and VII	Methanolic	[21]
	Physagulin D and coagulin Q	Methanolic	[101]
Fruits	Linoleic acid, palmitic acid, tetracosanoic acid, elaidic acid, and oleic acid	Oils	[95]
	Withanamides A-I	Methanolic	[98]
Stem bark	Withasomnilide, somniferanolide, somniferawithanolide, withasomniferanolide, and somniwithanolide	Ethanolic	[70]
Whole plant	Withaniol, acylsteryl glucosides, starch, reducing sugars, hantreacotane, ducitol, aspartic acid, proline, tyrosine, alanine, glycine, glutamic acid, cystine, tryptophan, withaniol, starch, acylsteryl glucosides, hantreacotane, ducitol	Methanolic	[20,21,34]
	6*α*-chloro-5*β*,17*α*-dihydroxywithaferin A 6*α*-chloro-5*β* hydroxywithaferin A, (22R)-5*β*-formyl-6*β*,27-dihydroxy-1-oxo-4-norwith-24-enolide, withaferin A, 2,3-dihydrowithaferin A, 3-methoxy-2,3-dihydrowithaferin A, 2,3-didehydrosomnifericin, withanone, withanoside IV, and withanoside X	Aqueous, methanolic	[61]
	Withanone and tubacapsenolide F	Aqueous	[54]
	Withasomniferin-A and iso-sominolide, and sominone	Ethanolic	[64,65]
	Viscosalactone B	Alcoholic	[73]
Aerial parts	27-acetoxy-4β,6α-dihydroxy-5β-chloro-1-oxowitha-2,24-dienolide), along with diepoxy withanolide	Methanolic	[60]

**Table 3:** Traditional medicinal applications of various parts of *Withania somnifera*

Plant part used	Uses	References
Roots	Treatment of asthma, bronchitis, leucoderma, tuberculosis, liver problems, heart disorders, and arthritis Act as an antibacterial, antitumor, antioxidant, immunomodulatory, and neurotic regenerator Show adaptogenic activity, nootropic effect, hypothyroid activity, herbicidal potential, abortifacient astringent, aphrodisiac, and emmenagogue,	[7,85,87,94, 117-122,123-130,131-133]
Leaves	Treatment of ulcers, painful swelling, external pains, syphilis, hemorrhoids, eyesores, boils, and edema Act as aphrodisiac, anti-inﬂammatory, diuretic, hepatoprotective, anti-arthritic, anti-cancerous, and pesticidal	[118,119,134-147]
Seeds	Act as a diuretic, narcotic, and hypnotic	[148]
Fruits	Treatment of ulcer and tuberculosis Act as antihelmintic	[40,149]
Leaves, roots, and stem	Act as antibacterial, antitumor, and herbicidal	[150-154]
Whole plant	Act as an antidote, insecticidal, larvicidal, antioxidant, immunomodulatory, neurotic regenerator, adaptogenic hepatoprotective, and cardioprotective	[11,55,155-163]

**Table 4:** Bactericidal potential of various parts of *Withania somnifera*

Plant part used	Nature of extract	Bacteria studied		Method adapted	MIC/ IC_50_	Standard Drug	References
		Gram-positive	Gram-negative				
Roots	Monomeric glycoprotein	*C. michiganensis subsp. Michiganensis* *B. Subtilis*	*P. fluorescens*	Paper diffusion method	10 *µ*L	-	[123]
	Aqueous, chloroform	*B. Subtilis*	*P. aeruginosa* *E. aerogens*	Disc diffusion method	3.75 mg/mL	Gentamycin	[150]
	Aqueous, DMSO extract fractionated into methanol, ethanol, butanol fractions	*S. aureus*	*-*	Disc diffusion method	100 mg/mL	Gentamycin	[152]
	Methanolic extract		*E. coli* Enterococcus sp	Disc diffusion method	10 *μ*g/mL	-	[97]
	Methanol	S. aureus	E. coli	Disc diffusion method	10-100 *μ*g/mL	Streptomycin sulfate	[166]
	Methanol, ethyl acetate, aqueous	-	S. typhimuriuma E.coli S. aureus	Disc diffusion method	20 mg/mL	Chloramphenicol	[124]
	Methanol, *n*-hexane, diethyl ether	-	S. typhimuriuma *E.coli*	Disc diffusion method	0.1 mg/mL	Tibrim	[165]
	Acetone, methanol, ethanol, chloroform	S. aureus	K. pneumonia	Disc diffusion method	50 *μ*g	Tetracycline, vancomycin, Sulphamethoxazole, trimetoprine, Impenem	[168]
Leaves	Glacial acetic acid, toluene	-	*P. mirabilis* *K. Pneumonia* *A. tumefaciens*	Disc diffusion method/ Serial dilution method	0.469-7.5 mg/mL	Gentamycin	[151]
	Methanol, ethyl acetate, aqueous	-	S. typhimuriuma E.coli S. aureus	Disc diffusion method	20 mg/mL	Chloramphenicol	[124]
	Methanol, *n*-hexane, diethyl ether	-	S. typhimuriuma *E.coli*	Disc diffusion method	0.1 mg/mL	Tibrim	[165]
	Methanol	S. aureus	E. coli	Disc diffusion method	10-100 *μ*g/mL	Streptomycin sulfate	[166]
	Methanol	S. aureus Enterococcus Sp	-	Disc diffusion method	1-2 mg/mL	-	[167]
Bark	Methanol	S. aureus	E. coli	Disc diffusion method	10-100 *μ*g/mL	Streptomycin sulfate	[166]

**Table 5:** Antifungal activity of different extracts of *Withania  somnifera*

Plant part used	Nature of extract	Fungal Strains	IC_50 _value	Standard drug	References
Roots	Monomeric glycoprotein	*A. flavus, A. niger, A. nidulans, A. flaviceps, A. alternate, A. carthami, F. oxysporum, * *F. verticilloides *	20 µg	Bavistin	[123]
	Aqueous, chloroform	*A. flavus*	0.938- 15 mg/mL	Ketoconazole	[150]
	Methanol	*A. flavus, D. turcica, F. verticillioides*	100 µg	Nystatin	[166]
	Aqueous, organic solvent	*F. crown*	100 mg/100 mL	-	[169]
Stem	Aqueous, organic solvent	*F. crown*	100 mg/100 mL	-	[169]
Leaves	Aqueous, organic solvent	*F. crown*	100 mg/100 mL	-	[169]
	Glacial acetic acid and toluene extract	*A. tumefaciens* *, A. niger*	0.938-15 mg/mL	Ketoconazole	[151]
	Methanol	*A. flavus, D. turcica, F. verticillioides*	100 µg	Nystatin	[166]

**Table 6:** Pharmacological attributes of various parts of various parts of *Withania somnifera*

**Pharmacological activity **	**Plant part used**	**Nature of extract **	**Dose of extract **	**Model animal **	**References **
Adaptogenic	Roots	Aqueous	25-50 mg/kg of body weight	Rats	[87]
		Ethanolic	23 mg/kg of body weight	Mice	[233]
		Alcoholic	12-25 g/kg of body weight	Albino rats	[51]
Anti-inflammatory	Roots	Powder	1000 mg/kg of body weight	Wistar rats	[182]
		Powder	600 mg/kg	Rats	[145]
	Leaves	Leaf powder	12-25 mg/kg	Albino rats	[43]
		Alcoholic	1 g/kg	Rats	[141]
	Whole plant	Methanolic	69-111 µg/mL	Lab assay	[182]
		Chloroform	76 to 132 µg/mL	Lab assay	[182]
		Ethanolic	764 mg/kg	Rats	[183]
	Roots	Aqueous	1000 mg/kg	Rats	[143]
Anti-stress	Seeds	Defatted alcoholic	100 mg/kg	Mice	[229]
	Whole plant	Alcoholic	100 mg/kg	Rats	[231]
Anti-tumor/cytotoxic	Leaves	Alcoholic	0.01-11.6 *μ*g/mL	Human cancer cell line (NCI-H460	[73]
			0.05-0.47 *μ*g/mL	Human cancer cell line (HCT-116)	[73]
			2.9-17.3 *μ*g/mL	Human cancer cell line (MCF-7 and SF-268)	[73]
	Leaves, roots, and stem	Ethanolic	50 g/100 mL	Human cancer cell lines	[153]
	Roots	Methanolic	0.43-1.48 µg/mL	Human cancer cell line	[100]
Antioxidant/ immunomodulatory	Fruit	Methanolic	0.5-1 *μ*g/mL 10 *μ*g/mL	Unilamellar vesicles model	[98]
	Roots	Aqueous	100 mg/kg	Mice and rabbits	[126]
		Alcoholic	100 mg/kg	Mice	[127]
		Alcoholic	20 mg/dose/animal	Mice	[81]
		Root powder	1000 mg/kg/day	Mice	[174]
	Whole plant	Aqueous	100 mg/kg/day	Albino rats	[214]
Neurotic regeneration	Root	Methanolic	10 mmol/kg of body weight	Mice	[11]
		Methanolic	Human neuroblastoma SH-SY5Y cell line	Human neuroblastoma SH-SY5Y cell line	[85]
		Aqueous and Methanolic	40 mg/kg of body weight	Rats	[128]
	Whole plant	Ethanolic	100, 200 and 300 mg/kg/body weight	Mice	[13]
		Methanolic	100 and 200 mg/kg	Rats	[228]
Neuro-protective	Leaf	Aqueous	140 g/kg for 15 days	Wistar rats	[189]
Anxiolytic and antidepressant	Roots	Methanolic	20 and 50 mg/kg	Rats	[209]
	Whole plant	Methanolic and aqueous	50 mg/kg		[235]
Hepatoprotective	Roots	Aqueous	10, 20, and 50 mg/kg of body weight	Rats	[208]
Nootropic and antigenotoxic	Root	Aqueous	50, 100, and 200 mg/kg	Mice	[128]
Cardioprotective	Whole plant	Hydro-alcoholic	25, 50, and 100 mg/kg	Rats	[162]
		Hydro-alcoholic	50 mg/kg	Wistar rats	[240]
Herbicidal	Roots and shoots	Aqueous, methanolic and *n*-hexane	5, 10, 15 and 20%	*P. minor*	[116]
			Foliar spray and soil application	*P. hysterophorus*	
			Soil application	*R. dentatus*	

## Conclusion and Future Prospects

Among the other medicinal plants of the family *Solanaceae*, the multipurpose plant *W. somnifera* has fascinated the researchers more, owing to the traditional therapeutic applications, nutraceutical potential, and pharmaceutical attributes. Folk healers cure various ailments such as cancer, arthritis, diabetes, eyesores, asthma, pyrexia, inflammations, hemorrhoids, ulcers, hepatitis, and wounds. Results of multiple preclinical trials such as antidepressant, antibacterial, anti-inflammatory, cardioprotective, anti-oxidant, antifungal, hepatoprotective, and hypoglycemic in animal models (mice, rats, and rabbits) are encouraging. The plant further requires the attention of phytochemists for the isolation of bioactives responsible for the biological activities so that new formulations are developed.

Literature has witnessed the anticancerous potential of the miraculous plant against various cancer cell lines, which is ascribed to the group of alkaloids called withanolides. Further efforts are neede for the isolation, purification, and commercial preparation of withanolides for therapeutic applications in human beings. Different extracts of the plant showed reasonable bactericidal and fungicidal potential. Separation of the phytochemicals responsible for the microbicidal potential is required for the development of new economical antibiotics with more significant therapeutic potential and lesser side effects. It will help to save the cost and lives of people.

It has been reported in the literature that the plant possessed natural anti-oxidants such as flavonoids, which strengthened the muscles and delayed the aging in clinical trials. However, extensive research is still required for the standardization and validation of the plant as an anti-aging agent. Non-steroidal anti-inflammatory drugs are usually used for the anti-nociceptive effect with side effects such as gastric ulcers. It has been discussed in the literature that plant extracts are beneficial in ulcers with anti-inflammatory potential. Further studies of the plant extracts will provide us the safer non-steroidal anti-inflammatory drugs with ulcer curing potential. The roots of the plant showed potential against obsessive-compulsive disorder. However, the active phytochemicals responsible for this activity are still unknown. Thus, there is a dire need to isolate phytochemicals for the development of commercial formulations for the obsessive-compulsive disorder. The plant parts have also been appraised for clinical trials such as male infertility, antianxiety, bone and muscle strengthening potential, hypolipidemic, and antidiabetic. Further clinical trials of different extracts of the plant are required for the development of economical and safer drugs. 

## Conflicts of Interest

We wish to confirm that there are no known conflicts of interest associated with the publication, and there has been no significant financial support for the work that could have influenced its outcome.
